# Development and psychometric properties of COVID-19 related Healthcare Student stress scale (CHSSS)

**DOI:** 10.1186/s40359-022-00778-9

**Published:** 2022-03-16

**Authors:** Nayereh Baghcheghi, Mehdi Mesri, Mahmood Karimi, Shoaleh Bigdeli, Hamid Reza Koohestani

**Affiliations:** 1 Social Determinants of Health Research Center, Saveh University of Medical Sciences, Saveh, Iran; 2 Department of Forensic Medicine, Faculty of Medicine, Baqiyatallah University of Medical Sciences, Tehran, Iran; 3 Department of Medical Education, School of Medicine, Center for Educational Research in Medical Sciences (CERMS), Iran University of Medical Sciences, Tehran, Iran

**Keywords:** COVID-19, Healthcare, Students, Stress, Scale, Psychometric properties

## Abstract

**Background:**

There is no valid and reliable tool to measure COVID-19 healthcare stress felt by healthcare students. A scale was developed to assess COVID-19 stress in healthcare students and its psychometrics was examined.

**Methods:**

This is a two phases mixed-method study including a qualitative stage consisting of student interview and literature review to develop content of the tool. In the quantitative stage, the psychometrics of the scale was examined in 2020–2021.

**Results:**

The COVID-19 related healthcare student stress scale (CHSSS) featured five factors including fear of catching coronavirus, social constraints, changes in education, non-compliance of health protocols and worrying news and overload information, which totally explained 51.75% of the total variance.

**Conclusion:**

Validity and reliability of CHSSS with 17 items were supported to measure COVID-19 stress in healthcare students as a self-assessment tool. Researchers can utilize this tool to assess COVID-19 stress in healthcare students and introduce policies and intervention especially designed for healthcare students.

**Supplementary Information:**

The online version contains supplementary material available at 10.1186/s40359-022-00778-9.

## Background

The COVID-19 caused pneumonia is now a global disease that is highly infectious and threatens public health [1, 2]. COVID-19 pandemics, affecting all the world, affect people not only physically but also psycho-social [3]. Expansion of epidemics intensifies or creates new stressors such as stress, fear and worry of oneself and loved ones’ health [4–6]. Lazarus and Folkman (1984) define stress as “a particular relationship between the person and the environment that is appraised by the person as tasking or exceeding his or her resources and endangering his or her well-being” [7]. The fear and anxiety stimulates the hypothalamus in the brain and increases secretion of cortisol from the membrane of adrenal glands, which in turn stimulates sympathetic nerves all over the body and prepares the body to deal with stressors in short term [5]. However, if the fear or stress and the body response (i.e. increased cortisol and sympathetic nerve stimulation) continue for long, they negatively affect the immune system and decrease the body’s capability to fight diseases including COVID-19 [8].

Healthcare students, in particular, are among the groups with high psychological vulnerability during the pandemics. The reasons for this is the highly competitive nature of their field of study, academic pressure, being in contact with patients, financial problems, and poor sleep quality, which can add to the psychological problems caused by the stress and anxiety [9, 10]. In addition, healthcare students during disease outbreak, experience a high risk of infection due to the higher risk of being exposed to virus during clinical training [11]. This means that these students suffer a higher level of anxiety mostly because of the concern of being infected and infecting their family members and loved ones [11]. Medical students have expressed high anxiety in clinical settings during pandemics [12]. A study in the US indicated that COVID-19 has significantly worsen mental health of university students; so that these individuals have to deal with a great deal of psychological pressures like anxiety, depression, post-traumatic stress disorder (PTSD), and eating problems [13].

The type and extent of stress in medical students is not the same as that in students in other fields [14–16]. Studies have shown that the level of stress in medical students is higher than that in non-medical students, which might be due to academic stress [17, 18]. In addition, consistent with the transactional model of stress, stress is a dynamic relational process that depends on the constant interaction between individual factors such as age and gender and situational factors. Therefore, the specific features of the target population in the development of tools and evaluate the sources of pressure perceived by them must be taken into account [19, 20].

The cause of stress in healthcare students is rooted in the fear of limitations and isolation as well as the probable changes in educational routines and everyday life. It is believed that these changes in academic life, being in contact with clinical settings, friends, university officials, instructors, and relatives can be a major source of stress in healthcare students. In general, regardless of the major changes in health professions students’ lives, there is no specially designed tool to assess and identify the specific sources of stress in these students during COVID-19 pandemic. This tool provides an early diagnosis of students with a high risk of having a significant psychological disease because of the pandemic. In addition, especially designed interventions can be introduced to foster wellbeing of these students. To fulfill this need, the present study proposed and validated a tool to measure sources of stress because of COVID-19 pandemic in healthcare students of Saveh University of Medical Sciences in Iran.

## Methods

### Design and setting

This mixed-method exploratory sequential research consists of two phases: (1) developing a scale (generating items), and (2) psychometrics examination of the developed scale. The study was carried out in 2020–2021 in Saveh University of Medical Sciences in Iran. In the first phase of the study, an inductive phase was followed by a deductive phase. In phase two, a psychometric evaluation of the developed tool and its validity and reliability assessment were performed.

### Qualitative study

Content analysis was performed as a part of phase one to shed light on the concept of COVID-19 stress in the health care students. The study participants were selected according to the maximum variation as to sex, age, study major, and academic level via purposive sampling that continued until data saturation. Totally, 12 interviews were carried out by the first author. The interviews were conducted at the university campus (classroom, office, and any place of convenience for the participants) in December 2020. The interviews were semi-structured and designed to examine the subjects’ experience with COVID-19 stress. The interviews were semi-structured and designed to examine the participants’ experiences about COVID-19 stress. Generally, the interviews took 30 to 50 min and data analysis was done following Graneheim and Lundman (2004) analysis method. The interviews were read and re-read several times to extract the meaning units and codes. The codes were categorized into different subcategories and categories based on their similarities and differences [21, 22]. To address trustworthiness, we used Lincoln and Guba’s criteria [23, 24].

### Item generation

The primary pool of items was developed based on the findings of the qualitative study and review of the available literature. The literature review continued till data saturation to find all items of the COVID-19 related healthcare student stress scale. The searched databases to find pertinent articles were Ovid, Science Direct, PubMed, and ProQuest. The search was conducted using keywords namely “development”, “COVID-19”, “psychometric”, “tool”, “student”, “healthcare”, “stress” and “scale.” The search yielded a tool with 92 items based on Likert’s five-point scale for psychometric evaluation.

### Psychometric evaluation

The psychometric properties including reliability, face validity, content validity, and construct validity were examined.

#### Face validity

Face validity was done qualitatively and quantitatively. As to qualitative face validity, 15 healthcare students were asked to express their opinions about difficulty level, relevance, and clarity of each scale item. Students were selected by convenience sampling method. As to quantitative face validity, the same participants rated each item in terms of importance based on a five-point Likert scale (not important = 1; relatively important = 2; moderately important = 3; fairly important = 4; and completely important = 5). To determine impact score of the items; relative frequency of the participants who scored items as 4 or 5 was multiplied by the mean importance score of the same item. Impact scores higher than 1.5 were considered appropriate [25].

#### Content validity

Content validity were evaluated by 10 experts specialized in psychology, psychiatry, public health and nursing. They included 6 professors, 2 associate professors, and 2 assistant professor. Experts were selected by convenience sampling method. Qualitative and quantitative approaches were used to determine content validity. Through qualitative validity, the experts were asked to comment such as the problematic understanding of the statements, the proportionality and proper relevance of the items with each other, possibility of ambiguity and misinterpretations regarding the statements or word meanings. The quantitative phase consisted of content validity index (CVI) and content validity ratio (CVR). In the CVR experts were asked independently to score the items on a three-point scale (‘necessary’, ‘useful but not necessary’, and ‘unnecessary’). Using Lawshe table, the items with CVR equal to 0.62 or higher were selected. As to CVI, 10 experts mentioned above rated each item based on relevance. To determine the item-level CVI (I-CVI), the number of experts who gave 3 or 4 points to each item was divided by the total number of experts. The CVI values equal to 0.78 or higher were considered satisfactory.

#### Construct validity

Construct validity was analyzed using exploratory factor analysis (EFA) and confirmatory factor analysis (CFA). An exploratory factor analysis (EFA) was conducted to investigate the latent constructs underpinning the scale and remove items with low factor loadings on common factors. Oblique rotation method was used in the EFA since the scale items were not completely unrelated to each other. Factors loading with eigenvalues equal to or greater than one were considered potential to retain. In each specific factor extracted only individual items loading at 0.4 or more were retained. To examine sampling adequacy for factor analysis, Bartlett test and Kaiser–Meyer–Olkin’s measure of sampling adequacy (KMO) were obtained. Data analysis was conducted in SPSS 16.0 (SPSS Inc., Chicago, IL, USA). Confirmatory factor analysis (CFA) was carried out in LISREL (version 8.8).

#### Sample size

According to EFA to determine construct validity, the study population consisted of all healthcare students in Saveh University of Medical Sciences (N = 578). The participants were selected from volunteers interested in participation (N = 306) using convenience sampling (response rate = 52.94%) in January 2021. An online questionnaire was developed and sent to students. Students’ emails were provided to the researchers by the university’s office. The inclusion criteria were undergraduate healthcare students, passed one year at least, and willing to take part in the study. The incomplete scales were excluded. The 17-item questionnaire was distributed electronically in the May of 2021, again among the students of the research environment using convenience sampling (570) and at this stage 426 students participated in the study (response rate = 74.34%). The data of this step were used for confirmatory factor analysis (CFA). In the second stage, in order to increase the motivation of participating in the study, prizes were awarded to three students by lottery. Possible reasons for some students’ non-participation in the study include lack of interest in the research topic or not checking email during the research period.

#### Reliability

Reliability of the scale was tested based on the internal consistency and stability criteria. To check the internal consistency of the scale, Cronbach’s alpha and theta coefficients were used. The stability of the scale was checked using test-retest method and the stability was determined based on intraclass correlation coefficient (ICC). Twenty students filled out CHESS twice in two consecutive weeks that was considered ad a fixed interval to avoid recall bias and sample changes.

## Results

Of the 92 items collected (see Additional file 1), 24 items were removed in the initial screening phase because they did not specifically fit the objectives of this study. After the initial screening process, 68 items remained in the scale. Next, based on experts opinion in qualitative validity phase, 40 items were removed from the list due to overlaps. None of the items were excluded in the face validity assessment and the 28 items were taken to CVR and CVI stage. In each of CVR and CVI assessment two more items were excluded respectively (4 items excluded in these phases).

Exploratory factor analysis (EFA) with oblique rotation was done and seven items were removed because they had a factor loading less than 0.4. Finally, 17 items remained in the scale.

The KMO score was 0.85, which indicates that the sample group was suitable for factor analysis. Moreover, the obtained Bartlett’s sphericity test was 1433, which was significant (P < 0.001) and the scale was suitable to determine the items and form factors. Exploratory factor analysis with oblique rotation revealed five factors with values higher than one (Table 1). The five-factor structure represented 51.75% of the total variance and the factors were labeled based on the items and content.

**Table 1:** Exploratory factor analysis results

Factor	Item	Factor loading
Fear of catching coronavirus	1. To what extent do you feel stress due to the risk of being infected by COVID-19 in public places?	0.86
	2. To what extent do you feel stress due to the risk of COVID-19 infection in training and clinical settings?	0.65
	3. To what extent do you feel stress due to the risk of COVID-19 infection in dormitories?	0.71
	4. To what extent do you feel stress due to the risk of COVID-19 infection in educational settings (laboratory, practice wards, workshops, etc.)?	0.86
	5. To what extent do you feel stress due to the risk of passing coronavirus on to the family members?	0.87
Social constraints	6. To what extent do you feel stress due to the constrained contact with family members and relatives?	0.65
	7. To what extent do you feel stress due to public and traffic limitations?	0.74
	8. To what extent do you feel stress due to limited contact with classmates and friends?	0.64
	9. To what extent do you fell stress due to limited contact with instructors?	0.62
Changes in education	10. To what extent do you feel stress due to attending online classes for theoretical courses?	0.85
	11. To what extent do you feel stress due to online tests?	0.83
	12. To what extent do you feel stress because of probable delay in educational processes (e.g. graduation) due to COVID-19 limitations?	0.88
Non-compliance of health protocols	13. To what extent do you feel stress due to non-compliance with health protocols by people in public places?	0.58
	14. To what extent due you feel stress due to non-compliance with health protocols in academic settings?	0.63
	15. To what extent do you feel stress due to lack of personal protection equipment?	0.85
Worrying news and information overload	16. To what extent do you feel stress due to worrying news and information overload in the media (TV, radio, papers, etc.) and social media?	0.64
	17. To what extent do you feel stress due to hearing about COVID-19 infection in your classmates or other students?	0.8

Mean scores and inter correlations among the factors of CHSSS are shown in Table 2.

**Table 2:** Correlation matrix between factors of CHSSS

	Mean(SD)	1	2	3	4	5
1.Fear of catching coronavirus	17.92 ± 4.9	1				
2.Social constraints	3.8 ± 9.82	0.49^ð^	1			
3.Changes in education	2.31 ± 10.4	0.39	0.46^ð^	1		
4.Non-compliance of health protocols	2.43 ± 8.61	0.54^ð^	0.47^ð^	0.29	1	
5.Worrying news and information overload	1.9 ± 5.15	0.61^ð^	0.51^ð^	0.38	0.46^ð^	1

^ð^P < 0.05

There were five items in factor 1 about fear of COVID-19 infection. There were four items in factor 2 related to social constraints. Factor 3 had three items about changes in educational routines. Factor 4 had three items about non-compliance with health protocols and factor 5 had two items related to worrying news and information overload.

Model fit indices are illustrated in Table 3, and goodness of fit of the model is shown by confirmatory factor analysis.

**Table 3:** Goodness of fit indices of CHSSS model

Fit Index type	Observed value	Acceptable value	Fit level
Relative χ2 fit index	1.08	< 3	Good fit
GFI	0.92	≥ 0.9	Good fit
IFI	0.98	≥ 0.9	Good fit
CFI	0.95	≥ 0.9	Good fit
NFI	0.91	≥ 0.9	Good fit
NNFI	0.97	≥ 0.9	Good fit
SRMR	0.04	≤ 0.05	Good fit
RMSEA	0.03	≤ 0.05	Good fit

The ICC of the tool was equal to 0.851 and that of the subscales was between 0.72 and 0.87, which supports the stability of the tool. Cronbach’s alpha was used to evaluate the internal consistency for scale. CHSSS showed good internal consistency on all the subscales and total scale (Table 4).

**Table 4:** Cronbach’s alpha for the scale and the subscales

Subscale	Cronbach’s alpha
Fear of catching coronavirus	0.94
Social constraints	0.88
Changes in education	0.91
Non-compliance of health protocols	0.83
Worrying news and information overload	0.89
Total	0.91

### Study participants

Regarding EFA, the participants consisted of 189 female (61.76%) and 117 male (38.24%) with age range of 18–30 years and mean age of 20.5 years (SD = 2.98). In terms of field of study participants were 90 nurse students, 72 operating room technology students, 62 anesthesia students (BSc), 40 prehospital emergency care students, and 42 midwifery students. With KMO = 0.85 and Bartlett’s test = 1433, adequacy of sample size and factorability were supported (p < 0.001). Participants in the confirmatory factor analysis phase included 259 female (60.8%) and 167 male (39.2%) with an age range of 20–39 years. In terms of field of study participants were 85 nursing students, 67 undergraduate operating room students, 57 undergraduate students in anesthesiology, 38 prehospital emergency care students, 37 midwifery, 35 occupational health, 37 environmental health, 39 public health and 31 health information technology students.

### Scoring the scale

The CHSSS was finalized with 17 items based on Likert five-point scale from zero (“Not at all stressful”) to four (“Extremely stressful”). The score range is from 0 to 68 and the lower score indicated the lower the stress and the higher score suggests a higher the stress. Therefore, the scores of the first factor (fear of catching coronavirus) is from 0 to 20, the second factor (social constraints) is from 0 to 16, third factor (changes in education) is from 0 to 12, the fourth factor (non-compliance of health protocols) is from 0 to 12 and the fifth factor (worrying news and information overload) is from 0 to 8. To compare between factors, the standardized mean (mean divided by the number of items) can be calculated. The items of the tool were translated into English by a bilingual experienced translator and then translated back into Persian by another translator. Then the original version was compared to the translated work. After examining differences, the final English version was obtained.

## Discussion

In the present study, two methods were used to check face validity (qualitative and quantitative face validity), three methods were used to check content validity (qualitative and quantitative approaches), two methods were used to determine construct validity (exploratory factor analysis and confirmatory factor analysis), and two methods were used to check reliability (cronbach’s alpha and test-retest method). Based on the results, validity and reliability of the tool to measure COVID-19 caused stress in healthcare students were supported. The tool is comprised of five sub-scales that measures distinct and correlated domains. The domains are (1) fear of catching coronavirus, (2) social constraints, (3) changes in education, (4) non-compliance of health care protocols, and (5) worrying news and information overload. Norbeck (1985) suggests that there are four minimum standards necessary for the adequate evaluation of a scale for use in research purposes. These standards should include at least one type of content validity, one type of construct (or criterion related) validity and two types of reliability testing [26].

The fear of catching coronavirus was the first factor with five items to measure perceived stress and the pertinent risk of cognition. The dimensions are consistent with the previous works on the major role of the fear of catching infection, the fear for one’s significant others (such as relatives and friends) to catch the disease, and fear of transmitting the disease to others [27, 28]. Compared to non-medical studies, medical students experience different situations such as clinical settings or patients’ bedside, which are highly stressful [29]. This is stronger during the current pandemic. As noted by students in the qualitative phase, a key source of stress for the students was the fear of catching infection during training and internship. Since these students are required to be in clinical settings to pass clinical and practical courses, this was the key stressor for them. However, it is notable that during COVID-19 pandemic, theoretical courses are held online and the majority of clinical and training courses are held with a few changes in the program.

Social constraints were the second factor with four items that covered the perceived stress of the study participants about traffic restrictions, and reduced visiting of relatives, and contacting colleagues and instructors. In fact, given the delay in the routines of the life of the students. In fact, given the delay in the routine of students [4, 30], this factor supported a greater perception of the dimensions of the changes in social constraints in university students.

The changes in education were the third factor with three items that created perceived stress due to disruption of routine educational methods. One of the stressors was holding on-line courses and exams during COVID-19 pandemic. Fawaz and Samaha (2021) reported that application of only e-learning based methods was the major cause of anxiety and depression in students because of the high load of work that was stress inducing [31]. In addition, COVID-19 pandemic has created changes in practical courses and final year students are required expand their program of study due to the delay in passing clinical courses required for graduation.

Non-compliance of health protocols was the fourth factor with three items that covered perceived stress due to non-compliance of health protocols in educational setting and community. Following health instructions is vital to slow down the spread of COVID-19 [32]. According to another study, there is a high rate of neglecting preventive measures during COVID-19 pandemic [32–34]. Given that the students have to travel inside of a city and between cities to attend their clinical courses, observing how the public does not follow health protocols in society and health centers, was a source of stress in the students that includes three items.

Worrying news and information overload was the fifth factor with two items. The fifth dimension is consistent with other studies that showed that social media has a significant effect on the spread of fear and panic due to COVID-19 pandemic. It has a potential negative effect on individuals’ mental health and psychological well-being [35].

As, human behavior has a strong impact on controlling the spread of COVID-19 pandemic, understanding the psychological responses in COVID-19 pandemic including anxiety and stress could inform intervention policies and the programs to combat COVID-19 by the public health officials and policy makers [3]. A study showed that college students are challenged during COVID-19 pandemic by psychological, social, and academic problems [13].

Therefore, dealing with the need for creating a specialized tool to find out the effect of COVID-19 in healthcare students [4, 36], the authors believe that the proposed tool can improve the chance of finding students with a higher risk of health complications due to the COVID 19 pandemic. Through this, these students can receive evidence-based and personalized interventions to have a better adjustment to the situation and to enjoy a higher wellbeing.

Stress can lower educational performance and facing of stressors, students might rely on smoking, drinking, and narcotic drugs. Students with a high level of clinical stress reported lack of self-confidence and low control over their educational process [37].

Taking into account the prolonged pandemic and harsh measures taken during the pandemic like lockdown, the negative effects of COVID-19 on healthcare professional students and their education is undeniable. The designed scale is recommended to be used for fields such as healthcare education and practice, and studies on healthcare students’ COVID-19 related knowledge about stress.

The tool can identify several stressors experienced by healthcare students during COVID-19 pandemic so that it can be used in planning and policy making about psychological interventions and improve mental health of students and increase personal capacities. Early diagnosis of stress and stressors and stress management can prevent mental problems and increase personal capacities. In addition, through expanding consultation programs and prioritizing high risk students, it is possible to improve their wellbeing and make the educational settings more efficient.

### Limitations

Despite these strengthens, there are also some limitations. Firstly, the study was conducted using online questionnaire, potentially limiting the enrollment in the study of those without Internet access. Given the requirement of quarantine and physical distancing during the COVID-19 pandemic, online surveys might be the only option. However, given that at the time of the study, the teaching method in Iranian universities was virtual, we believe that this limitation had little effect on the results. Secondly, participants were recruited only from one university of medical sciences in Iran. Further investigation on bigger and more representative samples is needed to confirm the results provided by the present study. Thirdly, although the CHSSS was designed for healthcare students, however, given that cultural and social variables may have potentially influenced the construct of the questionnaire, its generalizability to other students in other countries is unknown and must be tested. As with any new questionnaire, its use in different settings and in other countries will accumulate more robust evidence about its construct validity. Fourthly, given that participation in the study was voluntary and based on the response rate in the two phases of the study, the possibility of selection bias is another limitations in this study. Finally, it should be noted that in this study, many aspects of validity and reliability were not investigated (e.g., criterion validity, concurrent validity, predictive validity, convergent validity, discriminant validity).

## Conclusion

A stress scale with 17 items and five dimensions was developed to measure stress in healthcare students during COVID-19 pandemic. The psychometric properties of the scale were satisfactory, and the tool can be used to design personalized plans and to introduce support interventions for healthcare students.

## Supplementary Information

**Additional file 1**. The primary pool of items of scale.

## Data Availability

The datasets used and/or analysed during the current study are available from the corresponding author on reasonable request.
