# Supplementary material for: Development and psychometric properties of COVID-19 related Healthcare Student stress scale (CHSSS)
Source: BMC Psychol. 2022 Mar 16;10:68. doi: 10.1186/s40359-022-00778-9 (PMC8923965; doi:10.1186/s40359-022-00778-9)
Supplement: Supplementary file 1 — Additional file 1. The primary pool of items of scale. [file 40359_2022_778_MOESM1_ESM.docx]

**Appendice 1**. Primary pool of items of scale

| **Number** | **Items** | **From of the qualitative study/ literature review** | **impact score** | **Reason for exclude** |
| --- | --- | --- | --- | --- |
|  | I feel stress due to limited relationships with my university colleagues during this period of COVID-19 pandemic. | Qualitative study/ literature review |  | Overlap |
|  | Learning only in a virtual way during the Corona is stressful for me. | Qualitative study/ literature review | 1.98 | Exploratory factor analysis (EFA) |
|  | The relationships with my relatives during this period of COVID-19 pandemic is a concern for me. | Qualitative study / literature review |  | Overlap |
|  | I feel stress due to the risk of COVID-19 infection in dormitories. | Qualitative study | 2.58 | This item remained |
|  | I feel stressed due to the risk of contagion during this period of COVID-19 pandemic. | Qualitative study / literature review |  | Overlap |
|  | I feel stress due to non-compliance with health protocols in academic settings. | Qualitative study | 2.19 | This item remained |
|  | I'm afraid of contagion of COVID-19. | Qualitative study / literature review |  | Overlap |
|  | Academic workload in the Corona virtual training course has become more difficult. | Qualitative study | 1.98 | CVI |
|  | I felt irritated for not being able to go out with my friends. | Qualitative study / literature review |  | Overlap |
|  | The experience of virtual learning during the Corona is a concern for me. | Qualitative study | 2.19 | Exploratory factor analysis (EFA) |
|  | I become irritated because of social distancing maintained due to COVID-19. | Qualitative study / literature review |  | Overlap |
|  | I feel stress due to hearing about COVID-19 infection in my classmates or other students. | Qualitative study | 2.79 | This item remained |
|  | I felt sleep disturbances due to COVID-19. | Qualitative study / literature review |  | Non-specificity with research objectives (initial screening) |
|  | One of my concerns is the difficult travel conditions due to traffic restrictions. | Qualitative study |  | Overlap |
|  | I complained about having headaches, body pain, and feeling of faintness or dizziness due to COVID-19. | Literature review |  | Non-specificity with research objectives (initial screening) |
|  | I got disturbing nightmares or dreams due to COVID-19. | Literature review |  | Non-specificity with research objectives (initial screening) |
|  | I feel stress due to condition of social isolation imposed during this period of COVID-19 pandemic. | Qualitative study /literature review |  | Overlap |
|  | Not following the protocols by some patients and their relatives in the hospital environment is stressful for me. | Qualitative study |  | Overlap |
|  | I got worried after getting news and updates regarding the COVID-19. | Qualitative study /literature review |  | Overlap |
|  | I felt nervous about not being able to meet the deadline for virtual homework submissions. | Qualitative study/ literature review |  | Overlap |
|  | I felt stressed out with online task submissions. | Qualitative study | 2.19 | Exploratory factor analysis (EFA) |
|  | I felt stressed due to the excess load of task completion. | Qualitative study | 1.98 | Exploratory factor analysis (EFA) |
|  | I feel stress because of probable delay in educational processes (e.g. graduation) due to COVID-19 limitations. | Qualitative study | 2.4 | This item remained |
|  | I upset while thinking about my future academic life due to COVID-19. | Literature review | 2.19 | CVI |
|  | I worried about the impact of the pandemic on my educational processes or career prospects. | Qualitative study /literature review | 2.19 | CVR |
|  | I feel stress due to lack of personal protection equipment. | Qualitative study /literature review | 2.4 | This item remained |
|  | I am worried about catching the virus. | Qualitative study /literature review |  | Overlap |
|  | I am worried that I can’t keep my family safe from the virus. | Qualitative study /literature review |  | Overlap |
|  | I am worried that our healthcare system won’t be able to protect my loved ones. | Qualitative study /literature review |  | Overlap |
|  | I am worried our healthcare system is unable to keep me safe from the virus. | Qualitative study /literature review |  | Overlap |
|  | I felt irritated for not being able to go out with my family. | Qualitative study /literature review |  | Overlap |
|  | I felt frustrated for not being able to meet friends. | Qualitative study /literature review |  | Overlap |
|  | I am worried that basic hygiene (e.g., hand washing) is not enough to keep me safe from the virus. | Qualitative study /literature review |  | Overlap |
|  | I feel stress due to worrying news and information overload in mobile social media. | Qualitative study |  | Overlap |
|  | I am worried that social distancing is not enough to keep me safe from the virus. | Qualitative study /literature review |  | Overlap |
|  | I am worried that grocery stores will close down. | Literature review |  | Non-specificity with research objectives (initial screening) |
|  | I feel stress due to worrying news and information overload in the media and social media. | Qualitative study /literature review | 1.98 | This item remained. |
|  | I am worried about grocery stores running out of cleaning or disinfectant supplies. | Literature review |  | Non-specificity with research objectives (initial screening) |
|  | I am worried due to the risk of COVID-19 infection in training and clinical settings. | Qualitative study | 2.79 | This item remained |
|  | I am worried about grocery stores running out of water. | Literature review |  | Non-specificity with research objectives (initial screening) |
|  | I feel stress due to public and traffic limitations |  | 2.19 | This item remained |
|  | I feel stress due to online tests. |  | 2.4 | This item remained |
|  | I am worried about pharmacies running out of prescription medicines. | Literature review |  | Non-specificity with research objectives (initial screening) |
|  | I am worried that foreigners are spreading the virus in my country. | Literature review |  | Overlap |
|  | If I went to a restaurant that specialized in foreign foods, I’d be worried about catching the virus. | Literature review |  | Non-specificity with research objectives (initial screening) |
|  | I feel stress due to non-compliance with health protocols by people in public places. | Qualitative study | 1.98 | This item remained |
|  | I am worried about coming into contact with foreigners because they might have the virus | Literature review |  | Non-specificity with research objectives (initial screening) |
|  | If I met a person from a foreign country, I’d be worried that they might have the virus | Literature review |  | Non-specificity with research objectives (initial screening) |
|  | If I was in an elevator with a group of foreigners, I’d be worried that they’re infected with the virus | Literature review |  | Overlap |
|  | I am worried that foreigners are spreading the virus because they’re not as clean as we are | Literature review |  | Non-specificity with research objectives (initial screening) |
|  | I am worried that if I touched something in a public space (e.g.,handrail, door handle), I would catch the virus | Qualitative study /literature review |  | Overlap |
|  | I feel stress due to the risk of passing coronavirus on to the family members | Qualitative study /literature review | 3 | This item remained |
|  | I am worried that if someone coughed or sneezed near me, I would catch the virus | Qualitative study /literature review |  | Overlap |
|  | It is difficult to focus on an online learning session | Qualitative study | 2.58 | CVR |
|  | I am worried that people around me will infect me with the virus. | Qualitative study /literature review |  | overlap |
|  | I am worried about taking change in cash transactions. | Literature review |  | Overlap |
|  | I am worried that I might catch the virus from handling money or using a debit machine. | Literature review |  | Overlap |
|  | Numerous negative news of Covid 19 disease puts a lot of stress on me. | Qualitative study |  | Overlap |
|  | Thinking about Corona has made me lose my appetite | Literature review |  | Non-specificity with research objectives (initial screening) |
|  | I am worried that my mail has been contaminated by mail handlers | Literature review |  | Non-specificity with research objectives (initial screening) |
|  | It makes me uncomfortable to think about Corona | Literature review |  | Overlap |
|  | I had trouble concentrating because I kept thinking about the virus | Literature review |  | overlap |
|  | I feel stress due to limited contact with classmates and friends | Qualitative study | 2.19 | This item remained |
|  | Disturbing mental images about the virus popped into my mind against my will | Literature review |  | Non-specificity with research objectives (initial screening) |
|  | I had trouble sleeping because I worried about the virus. | Literature review |  | Non-specificity with research objectives (initial screening) |
|  | I feel stress due to the risk of COVID-19 infection in skill lab. |  |  | Overlap |
|  | I feel stress due to attending online classes for theoretical courses. | Qualitative study | 2.4 | This item remained |
|  | I thought about the virus when I didn’t mean to. | Literature review |  | Non-specificity with research objectives (initial screening) |
|  | Reminders of the virus caused me to have physical reactions, such as sweating. | Literature review |  | Non-specificity with research objectives (initial screening) |
|  | I feel stress due to the risk of being infected by COVID-19 in public places. | Qualitative study /literature review | 2.4 | This item remained |
|  | I had bad dreams about the virus. | Literature review |  | Non-specificity with research objectives (initial screening) |
|  | My heart races or palpitates when I think about getting Corona | Literature review |  | Non-specificity with research objectives (initial screening) |
|  | I worry about coronavirus from other people in a crowded place. | Qualitative study /literature review |  | Overlap |
|  | Through online learning, education has become a heavy burden. | Qualitative study | 2.19 | Exploratory factor analysis (EFA) |
|  | I worry about infection if other people don't wear masks. | Qualitative study /literature review |  | Overlap |
|  | There is a lot of stress before an online learning session or online test due to an internet connection. | Qualitative study | 2.58 | Exploratory factor analysis (EFA) |
|  | I feel stress due to the risk of COVID-19 infection in laboratory (for education). | Qualitative study |  | Overlap |
|  | I am worried about grocery stores running out of food. | Literature review |  | Non-specificity with research objectives (initial screening) |
|  | I feel stress due to the risk of COVID-19 infection in educational settings. | Qualitative study | 2.58 | This item remained |
|  | I'm worried about possible delays in my graduation. | Qualitative study |  | Overlap |
|  | I feel stress due to the constrained contact with family members and relatives. | Qualitative study | 2.58 | This item remained |
|  | I fell stress due to limited contact with instructors. | Qualitative study /literature review | 1.98 | This item remained |
|  | I'm worried about getting the Coronavirus while traveling on public transport. | Qualitative study |  | Overlap |
|  | I cannot sleep because I’m worrying about getting coronavirus-19. | Literature review |  | Non-specificity with research objectives (initial screening) |
|  | When watching news and stories about coronavirus-19 on social media, I become nervous or anxious. | Qualitative study /literature review |  | Overlap |
|  | I am afraid of catching COVID-19 through clinical practice. | Qualitative study |  | Overlap |
|  | I have trouble relaxing when I think about COVID-19. | Literature review |  | Overlap |
|  | I am worried about grocery stores running out of cold or flu remedies. | Literature review |  | Non-specificity with research objectives (initial screening) |
|  | My body trembles when I think about Corona. |  |  | Non-specificity with research objectives (initial screening) |
|  | My hands become clammy when I think about Corona | Literature review |  | Non-specificity with research objectives (initial screening) |
|  | Understanding online content is difficult to learn. | Qualitative study /literature review | 1.98 | Exploratory factor analysis (EFA) |
|  | I am worried that if I touched something in a public space (e.g., handrail, door handle), I would catch the virus. | Qualitative study /literature review |  | Overlap |
